# Supplementary material for: Mutations in PCYT2 disrupt etherlipid biosynthesis and cause a complex hereditary spastic paraplegia
Source: Brain. 2019 Oct 22;142(11):3382–97. doi: 10.1093/brain/awz291 (PMC6821184; doi:10.1093/brain/awz291)
Supplement: awz291_Supplementary_Data [file awz291_supplementary_data.zip › awz291-Suppl_data/Supplementary_Data4.zip!/Supplementary information 4 - plasma lipidomics.zip!/Plasma lipidomics/Control vs Patient3b/Statistical plots and data/OPLS-DA.pdf]

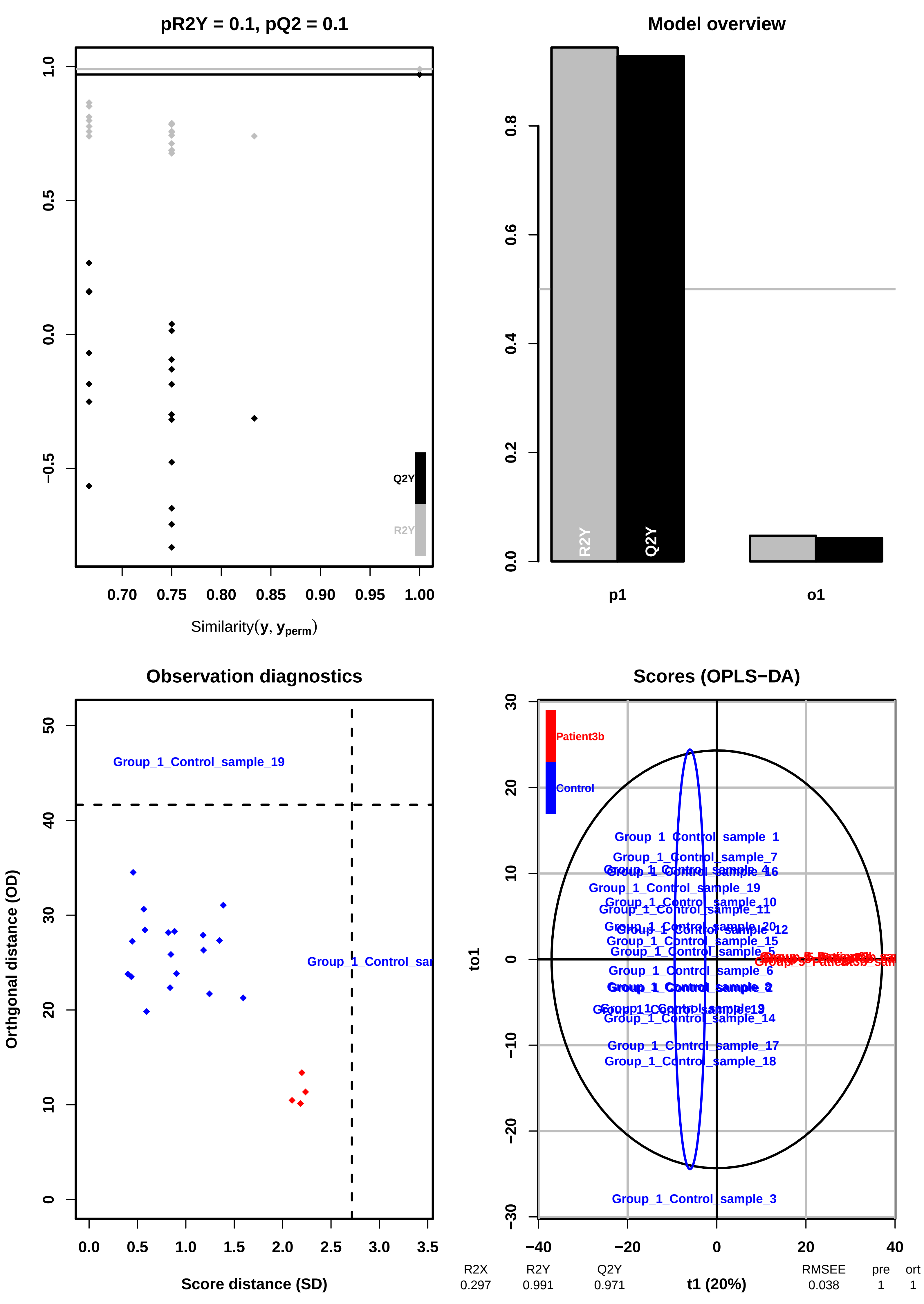

pR2Y = 0.1, pQ2 = 0.1
Similarity(y, yperm)
Q2Y
R2Y
Model overview
R2Y
Q2Y
p1
o1
Observation diagnostics
Group_1_Control_sample_19
Score distance (SD)
Orthgonal distance (OD)
Scores (OPLS−DA)
Patient3b
Control
Group_1_Control_sample_1
Group_1_Control_sample_7
Group_1_Control_sample_19
Group_1_Control_sample_10
Group_1_Control_sample_11
Group_1_Control_sample_15
Group_1_Control_sample_5
Group_1_Control_sample_6
Group_1_Control_sample_14
Group_1_Control_sample_17
Group_1_Control_sample_18
Group_1_Control_sample_3
t1 (20%)
to1
R2X 0.297
R2Y 0.991
Q2Y 0.971
RMSEE 0.038
pre 1
ort 1
